# A Tiled Ultrasound Matrix Transducer for Volumetric Imaging of the Carotid Artery

**DOI:** 10.3390/s22249799

**Published:** 2022-12-14

**Authors:** Djalma Simões dos Santos, Fabian Fool, Moein Mozaffarzadeh, Maysam Shabanimotlagh, Emile Noothout, Taehoon Kim, Nuriel Rozsa, Hendrik J. Vos, Johan G. Bosch, Michiel A. P. Pertijs, Martin D. Verweij, Nico de Jong

**Affiliations:** 1 Laboratory of Medical Imaging, Department of Imaging Physics, Delft University of Technology, 2628 CJ Delft, The Netherlands; 2 Electronic Instrumentation Laboratory, Delft University of Technology, 2628 CD Delft, The Netherlands; 3 Department Biomedical Engineering, Thoraxcenter, Erasmus Medical Center, 3015 GD Rotterdam, The Netherlands

**Keywords:** ultrasound transducer, matrix array, lead zirconate titanate (PZT), application-specific integrated circuit (ASIC), high-frame rate, three-dimensional (3D), ultrasound imaging, carotid artery

## Abstract

High frame rate three-dimensional (3D) ultrasound imaging would offer excellent possibilities for the accurate assessment of carotid artery diseases. This calls for a matrix transducer with a large aperture and a vast number of elements. Such a matrix transducer should be interfaced with an application-specific integrated circuit (ASIC) for channel reduction. However, the fabrication of such a transducer integrated with one very large ASIC is very challenging and expensive. In this study, we develop a prototype matrix transducer mounted on top of multiple identical ASICs in a tiled configuration. The matrix was designed to have 7680 piezoelectric elements with a pitch of 300 μm × 150 μm integrated with an array of 8 × 1 tiled ASICs. The performance of the prototype is characterized by a series of measurements. The transducer exhibits a uniform behavior with the majority of the elements working within the −6 dB sensitivity range. In transmit, the individual elements show a center frequency of 7.5 MHz, a −6 dB bandwidth of 45%, and a transmit efficiency of 30 Pa/V at 200 mm. In receive, the dynamic range is 81 dB, and the minimum detectable pressure is 60 Pa per element. To demonstrate the imaging capabilities, we acquired 3D images using a commercial wire phantom.

## 1. Introduction

Carotid arteries are major blood vessels located on both sides of the neck that supply the head and brain with oxygen and nutrients. Carotid artery disease, which is referred to as atherosclerosis or stenosis, occurs when fatty deposits (plaques) clog the carotid artery [1]. The blockage of the carotid arteries is a frequent source of stroke, a medical emergency that occurs when the blood supply to the brain is interrupted or seriously reduced [2]. Assessing the progression of atherosclerosis in the carotid artery is very useful for risk stratification, evaluation of patient response to medical interventions, evaluation of new risk factors, genetic research, and quantification of the effects of new therapies [3,4]. The assessment of the carotid plaque state is commonly performed with ultrasound imaging for the purpose of medical diagnosis [5]. 

With conventional two-dimensional (2D) ultrasound imaging, the assessment of the plaque is based on multiple 2D images, which are mentally combined by the operator to form a subjective impression of the three-dimensional (3D) vessel structure. Using this approach, accurate assessment of the plaque progress is difficult and highly dependent on the skills and experience of the sonographer [6]. This requires the reproduction of the same imaging plane at later times, which is difficult and sometimes impossible due to the restrictions imposed by the patient’s anatomy or position. Moreover, quantitative estimation of the plaque volume from a 2D ultrasound is based on measurements of height, width, and length in different orthogonal views for ideal shapes (e.g., ellipsoidal), which are prone to error [7]. A 3D ultrasound has the potential for accurate quantitative monitoring of the changes in plaque volume and might be vital for therapy assessment [2,4,8,9]. In carotid artery diagnosis, measurement of blood flow and plaque surface motion are important parameters [10]. For accurate analysis of the dynamics of the blood flow, 3D vector velocities at a high frame rate are necessary. Two-dimensional methods do not provide a realistic picture of the actual flow and do not provide information about the out-of-plane velocity component [11]. Thus, these 3D phenomena can only be assessed correctly with high frame rate 3D ultrasound imaging.

Going from 2D to 3D high-frame rate ultrasound imaging is challenging. While mechanically swept or free-hand scanning techniques using a linear (i.e., one-dimensional) transducer array might suffice for low-frame rate applications, a 2D matrix transducer array is necessary for high-frame rate applications [7]. This matrix transducer should cover a sufficiently large aperture (>400 mm^2^) and its element pitch should preferably be smaller than half of the wavelength (λ) in both directions to avoid grating lobes. The combination of small elements and a large aperture results in a very large number of transducer elements (in the order of thousands) [12,13,14]. It is possible to build a matrix array with such a vast number of elements, but making electrical connections to all the elements is a great challenge [15]. Various techniques have been proposed to reduce the complexity of fully populated matrix arrays, such as sparse matrix arrays and row-column addressed matrix arrays. Sparse arrays can effectively reduce channel count and electronic complexity, and can perform high-frame rate volumetric imaging [16,17,18,19]. However, this type of array has two fundamental limitations, which are the lower signal-to-noise ratio (SNR) and higher clutter levels [20,21,22]. Row-column addressed arrays, on the other hand, can reduce the number of connections from *N*^2^ to 2*N* in a matrix array consisting of N × N elements [23,24,25]. However, the inherent drawbacks of this transducer are the more complex read-out sequences and the lower frame rate, which is limited due to switching [21].

As an alternative, application-specific integrated circuits (ASICs) can be directly integrated with the matrix array to reduce the number of electrical connections, allowing large-element-count transducers to be used with traditional 128- to 256-channel systems and probe cables [26,27]. With this approach, the channel reduction can be done in multiple ways depending on the intended application, for example by channel multiplexing, sub-aperture beamforming, in-probe-digitization, or time-division multiplexing [28,29,30]. Besides channel reduction, an ASIC can also perform amplification of the received signals to prevent attenuation due to the loading of the cables connecting the ASIC to the imaging system [31,32]. These advantages make matrix arrays with in-probe electronics an attractive technology for 3D high-frame rate ultrasound imaging, although this comes at the cost of a more complex and costly developing process. Examples of commercially available matrix array probes with a large element count and integrated ASIC include the xMATRIX technology from Philips [33], the iQ+ technology from Butterfly [34,35], and the 4G CMUT technology from Fujifilm [36].

We have previously presented a first-generation matrix transducer array made of lead zirconate titanate (PZT), designed for high-frame rate, 3D imaging of the carotid arteries that was built directly on top of an ASIC [14,31]. Since building one single ASIC large enough to cover the carotid bifurcation is very challenging and expensive, we opted for a tiling approach in which multiple small identical ASICs were tiled together in both the lateral and elevation direction to form a larger array. A single ASIC contained 24 × 40 element-level circuits that consisted of transmit (TX) switches, receive (RX) switches, and control logic. The ASIC architecture could accommodate nine arbitrary TX/RX patterns in the provided memories. The architecture allowed the matrix to operate like an electronically translatable array and we have previously shown how to achieve a high frame rate with such a transducer [37]. The layout of element-level circuits was matched to the element pitch of the matrix array, which was 150 μm in both directions. By using 10 × 2 tiled ASICs, a probe with an aperture of 36 mm × 12 mm consisting of 19,200 elements (240 rows and 80 columns) could be constructed; however, at that time, we only presented a 4 × 2 tiled design, which was too small for carotid artery imaging. Although the functionality of this matrix transducer was successfully demonstrated in a 3D imaging experiment [14], the probe had limitations in terms of transmit voltage, cable count, and the number of pre-programmed patterns, which needed to be tackled in the next generation of the probe.

In the second generation of the transducer, presented in this paper, we have developed a prototype to resolve the limitations present in the previous design. The new version contains 12 × 80 elements per ASIC that interface with a PZT matrix array of 300 μm × 150 μm. Due to this, we only need to tile the ASICs in one direction instead of two directions, which makes the alignment during manufacturing much easier. In addition, the larger pitch overcomes electronics space limitations in the pitch-matched configuration, which allows increasing the TX pulse voltage from 30 V to 65 V. Additionally, the number of pre-programmed arbitrary patterns is increased to 20. Finally, undesired transmit signals that originated from the TX-to-RX mode switching (and vice versa) are significantly reduced in the new version of the ASIC [15].

This paper describes the development of the prototype tiled matrix transducer that is based on the second-generation ASIC described above. The goal of this work is to demonstrate the feasibility of using the tiling approach to build a sufficiently large aperture, and to evaluate the potential of using the prototype for 3D imaging of the carotid artery. In so doing, we will discuss the design, fabrication process, and extensive characterization of the transducer that was targeted to have 7680 piezo elements built on top of 8 × 1 tiled ASICs. We also performed volumetric imaging of a wire phantom to demonstrate the imaging capabilities of the prototype.

## 2. Materials and Methods

### 2.1. Design Choices

Volumetric imaging currently cannot attain the same frame rate, resolution, and image quality as in 2D, at least not all at the same time. The main issue is that with a large fully populated matrix array there are too many elements to control and read out at once. Therefore, trade-offs exist between frame rate, resolution, and image quality, which are different for the various types of matrix arrays discussed in the introduction. We have opted to use an ASIC to have a large field of view while still maintaining high frame rates. In this section, we discuss our design choices. 

**Figure 1:**
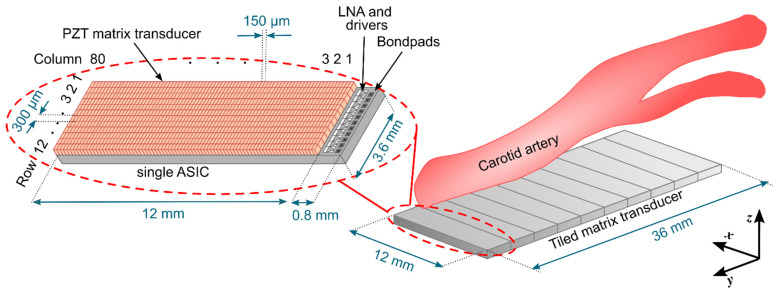
Schematic drawing of the envisioned full matrix transducer (**right**), together with a single ASIC transducer with PZT elements mounted on top (**left**).

The matrix transducer Is designed for imaging of the carotid bifurcation. We have chosen a center frequency of 7.5 MHz as this is recommended for carotid imaging applications [38]. For the aperture size, we aimed at about 40 mm × 15 mm as the carotid bifurcation is easily accessible and therefore allows for larger apertures. However, manufacturing such a large ASIC in one piece would be very expensive [39]. Thus, we opted to employ a tiled approach where we place multiple smaller and identical ASICs next to each other to create a larger aperture. The final aperture will be 36 mm × 12 mm, which consists of an array of 10 × 1 tiled ASICs of 3.6 mm × 12 mm each. A schematic drawing is shown in Figure 1.

A large aperture requires a large number of elements, but the number of available channels on an ultrasound system is limited. Research scanners like the ULA-OP [40] and the Verasonics Vantage [41] nowadays contain up to 256 channels. Our ASIC design requires separated TX and RX channels; therefore, we opted to limit ourselves to 128 transmit and 128 receive channels. However, since filling the total aperture with square elements with a pitch of 0.5 λ would require over 40,000 elements, and thus, over 40,000 channels, a significant degree of channel reduction is required.

As we are using ASICs, there are various ways to reduce the channel count. In our design, we make use of the fact that the fastest flow velocities in the carotid bifurcation are achieved along the long axis of the vessel, corresponding to the long side of the probe aperture (y-direction in Figure 1) and that the velocities in the direction of the short side (x-direction) are in comparison much slower. We can therefore use an asymmetric design where each row of the probe has a single transmit and receive channel and the columns can be enabled or disabled at will. This design represents an electronically translatable 1D array (translatable in the x-direction). Using this approach, assuming a pitch of 0.5 λ, the channel count could be reduced to 360 transmit and 360 receive channels, which would still be too high for contemporary research scanners. A way to further reduce the channel count is by increasing the element pitch in the y-direction to 300 μm, which corresponds to 1.5 λ. Such a large pitch can result in grating lobes and limited steering capability, yet it could be acceptable as we have chosen to make the aperture very large, and therefore high steering angles are not necessary. Another downside of having larger PZT elements is that they will not vibrate like a piston because the element width is much larger than 1.5 λ [42]. Instead, unwanted vibration modes will be generated, which significantly reduce the efficiency of the transducer [42,43]. Fortunately, the performance of elements having a width greater than 0.7 λ can be improved by subdicing the elements, as we have previously investigated through simulations [43] and experiments [44]. With the proposed pitch of 300 μm in the y-direction, only 120 TX and 120 RX channels are necessary, which satisfies our requirements.

### 2.2. Imaging Scheme 

**Figure 2:**
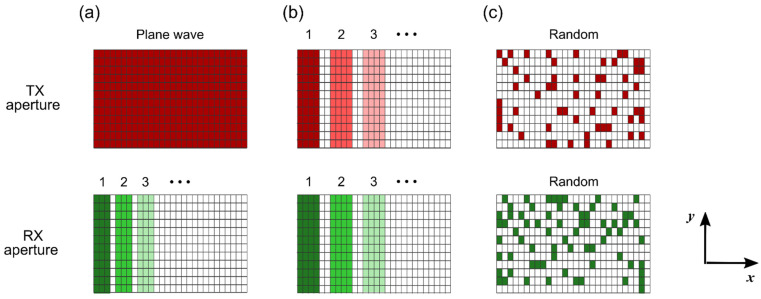
Example imaging schemes determined by different transmit and receive element configurations. (**a**) Plane-wave imaging. (**b**) Dynamic linear array. (**c**) Random pattern imaging.

Each imaging modality requires a different trade-off between spatial and temporal resolution: conventional B-mode requires the highest spatial resolution possible, Doppler imaging needs the best temporal resolution [25,45], and pulse wave imaging (i.e., wall motion imaging during the pulse wave in the blood vessels) requires a bit of both [3]. The designed ASIC allows for an arbitrary element group selection and thus supports different element configurations for different imaging purposes [31]. A few examples are outlined in Figure 2. For high-resolution B-mode imaging, the whole aperture can be used for transmission, while reception can take place column by column. For high-frame rate flow imaging, the transducer can operate like an electronically translatable fully addressable 1D array where a subset of the columns is used in transmit and receive, and these columns are translated between each TX/RX event. For other purposes, specific patterns or even pseudo-random selection are possible [31,46].

### 2.3. ASIC Design and Implementation

**Figure 3:**
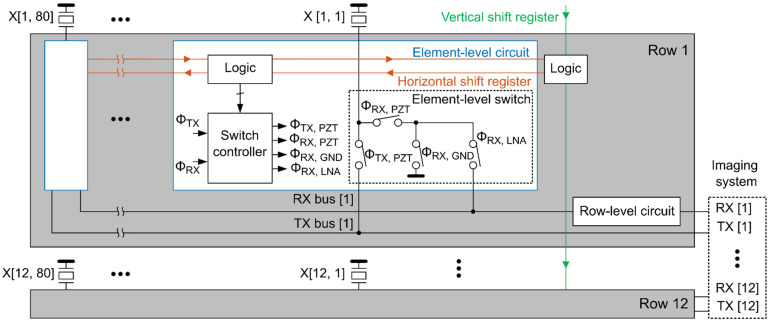
Block diagram of the architecture of a single ASIC.

Figure 3 shows the top-level architecture of the second-generation ASIC. Each row of the matrix has 80 elements that share row-level TX and RX buses to reduce the channel count by a factor of 80. Each transducer element has a programmable switching circuit that allows the element to be connected to the TX bus, the RX bus, both TX and RX buses (i.e., pulse-echo operation), or to the ground (i.e., disabled). The element-level circuitry fits in the 300 μm × 150 μm area occupied by the transducer element, allowing the PZT matrix to be integrated directly on top of the ASIC.

Each RX bus is associated with a shared row-level circuit consisting of a low-noise amplifier (LNA), a programmable gain amplifier (PGA), and a cable driver, which connects the output signal to a receive channel of the imaging system. The various combinations of LNA/PGA gain settings allow the achievement of a programmable gain ranging from −8.6 dB to 32.7 dB with an average step of 2.75 dB. The gain can be changed during the receive phase to make sure the signal level stays within the dynamic range of the ASIC. Each TX bus receives an externally generated high voltage transmit unipolar pulse with an amplitude of tens of volts. The control logic, which is programmed through horizontal and vertical shift registers, determines whether an element participates in a given TX/RX cycle; selects the element configuration for a specific imaging scheme; and sets the gain of the row-level circuit. More details about the current ASIC design can be found in our previous publication [15].

### 2.4. Acoustic Stack Design and Fabrication

#### 2.4.1. Stack Design

Our stack design was similar to what we have used previously for matrix transducers in our lab [3,14,31,37,47] and consisted of a PZT array built on top of an ASIC, a single matching layer, an aluminum ground foil, and a top protective layer. At the bottom of the PZT, there was a buffer layer that allowed for tolerances in dicing depths and electrically isolated neighboring elements. A major issue in the previous designs was the fact that the acoustic stack was mounted only through thin layers on top of the ASIC. This made fabrication easier, but due to the lack of damping between the PZT and the ASIC, a significant amount of energy was transmitted into the ASIC, which hardly attenuates the waves. This resulted in two effects: reflections from layers beneath the ASIC; and crosstalk between elements due to the propagation of lamb waves, which can be visible as extra peaks in the directivity pattern [47]. There are various approaches to reducing the effect of the ASIC on acoustic performance. Shabanimothlag et al. proposed to either lap down the thickness of the ASIC and place a standard acoustic backing behind the ASIC, or dice deep cuts into the ASIC [47]. These approaches work in simulation, but are difficult to realize in practice as the ASIC mainly consists of silicon, which is brittle and hence hard to process. All alternative methods use an interfacing surface layer such as epoxy between the ASIC and PZT to induce a quarter-wavelength mode and direct most of the acoustic energy from the PZT forward [48]. Wildes et al. proposed a high-impedance “dematching” layer (DML) that reduced the need for a high-attenuation backing layer [27]. They used anisotropic conductive adhesive and flex circuits to attach the acoustic stack to the ASIC. The disadvantage of this approach is that the dicing depth must be carefully controlled to cut through the DML and fully isolate the elements while not cutting through the flex circuit, which would damage the circuit traces. Wodnicki et al. proposed the use of an interposer conductive backing of pillars to connect the acoustic stack to the surface of the ASIC [49]. The interposer consisted of a 3D-printed acrylic frame that was filled with conducting and acoustically absorbing silver epoxy material. The thickness of the interposer backing was considerably large (4 mm) to ensure great attenuation. The downside of this approach is that the assembly of the interposer to the ASIC is a potential source of failures in electrical connections, and the size of the acrylic wall is limited by the resolution of the 3D printer.

**Figure 4:**
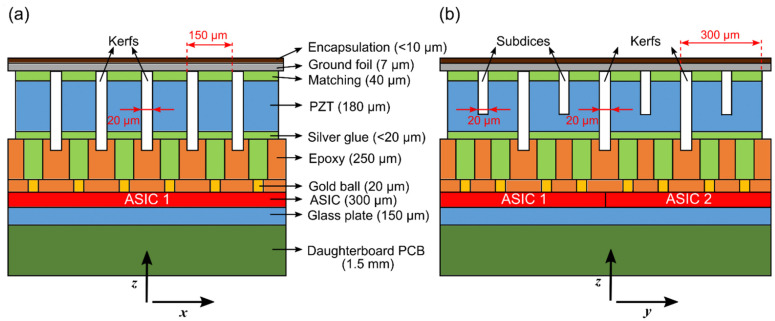
Overview of the acoustic stack (not drawn to scale). (**a**) Front view. (**b**) Side view. The numbers in parenthesis indicate the dimension in the z-direction (i.e., thickness).

In the current design, we have opted for an interposer layer that consists of pillars of silver glue and an epoxy material with high attenuation. The composite layer consists of a small channel of silver glue that electrically connects the PZT material to the ASIC and an epoxy that we previously used as a backing and has high attenuation. The thickness of the interposer was chosen such that it dampens the waves significantly, but not completely, as in the work of Wodnicki et al. With the chosen thickness, the interposer can be manufactured using conventional dice-and-fill methods. A schematic drawing of the designed acoustic stack with material information is presented in Figure 4. As seen, the interposer layer also serves as a buffer layer, which provides a margin for the dicing.

#### 2.4.2. Stack Fabrication

In our previous work [44], in which we investigated the effect of subdicing on a PZT matrix built on top of ASICs, we limited ourselves to the fabrication of a matrix transducer consisting of 4 × 1 tiled ASICs. The main difficulty lay in maintaining a flat surface of the stack over the whole area. The flatness of the stack surface is crucial for the dicing process because the dicing kerfs should be deep enough to guarantee the electrical and acoustical isolation between the elements, but shallow enough to not cut into the ASICs. Unfortunately, ASIC damage during the dicing process was a recurrent problem encountered in our previous attempts to fabricate the matrix.

In the current work, in order to minimize the risk of ASIC damage when dicing, we opted to manufacture a sample consisting of 8 × 1 tiled ASICs with a gap of one ASIC in the middle, i.e., the acoustic stack consists of two times 4 × 1 tiled ASICs with a gap of one ASIC in between the two. This is not the final version of the matrix transducer; however, this prototype is certainly relevant to verify the reliability of our manufacturing process, and to evaluate the functionality and performance of a prototype twice as large as our previous ones.

The transducer fabrication process starts with gluing the ASIC tiles onto a glass plate, which acts as a flat surface to guide the alignment of the ASICs and ensure that the ASICs have the same surface height. After this, gold balls are deposited on the ASIC pads (two bond pads are available per element to improve the connection stability). The gaps between the gold balls are filled with an electrically isolating epoxy material, which is then ground down until the gold balls are again exposed. The main role of this layer is to establish a mechanical buffer for dicing the interposer layer and to electrically isolate neighboring elements from each other. This buffer layer also provides the electrical connections from the ASIC bond pads to the interposer layer.

Next, the interposer is built by first depositing a thick layer of the non-conductive epoxy material. A 50 μm dicing blade is used to dice in one direction to expose the gold balls. These grooves are then filled with conductive glue and afterward, the excess material is ground away. Then, the dicing blade is used to dice in the other direction in between the gold balls, and these grooves are filled again by the non-conducting buffer material. Excess material is again ground away so that we end up with a flat top layer. 

Next in the process, a matching layer made of conductive glue is applied on top of the piezoelectric material (3203HD, CTS Corporation, Lisle, IL, EUA). Then, the stack consisting of PZT and the matching layer is glued on top of the interposer. After this, the acoustical stack is diced. Two types of cuts are used. The through-cuts are made to separate the PZT elements with 300 × 150 μm pitch, and they may partially extend into the interposer layer (see Figure 4). The subdicing cuts, on the other hand, only extend to about 70% of the pillar thickness. All the dicing/subdicing cuts are made using a 20 μm thick diamond blade and the dicing kerfs are not re-filled with any material, to minimize the acoustical crosstalk between the elements.

**Figure 5:**
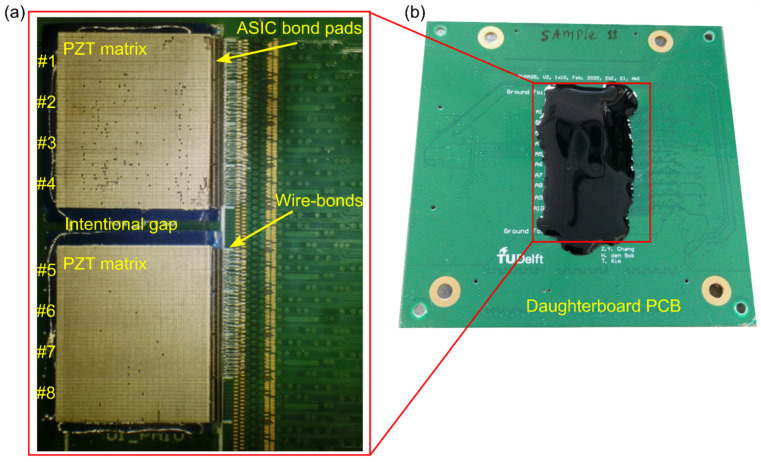
Photograph of the prototype transducer. (**a**) Fabrication of the PZT matrix with 96 × 80 (rows × columns) elements on top of 8 × 1 tiled ASICs. (**b**) The finished transducer on the daughterboard.

To finalize the matrix, a common ground electrode is made by gluing a 7 μm thick aluminum foil on top of the whole matrix array. Afterward, a thin layer of encapsulation material (AptFlex F7, Precision Acoustics Ltd., Dorchester, UK) is placed on top to prevent moisture from infiltrating the acoustic stack and thereby damaging the array. Photographs taken during and after the fabrication of the matrix transducer are shown in Figure 5.

#### 2.4.3. Electrical Connections

A daughterboard (see Figure 5b) was designed to hold the matrix transducer, and to provide the transmit, receive, power, and control signals to the ASIC. The electrical connections from the ASIC bond pads to the daughterboard bond pads were made with a bonding machine using 18 μm thick aluminum wire bonds. Ultraviolet curing glob top epoxy was applied over the bonding wires for protection. 

The daughterboard is connected to a motherboard by micro coaxial cable assemblies (Samtec, New Albany, IN, USA) to transfer the TX and RX data. The motherboard interfaces with a Verasonics imaging system (V1, Verasonics, Inc., Kirkland, WA, USA) via two connectors (DLM5-260PW6A, ITT Corporation, White Plains, NY, USA) such that it can be mounted directly on the Verasonics machine. An electronic matching network is provided in the transmit paths to minimize overshoot and undershoot of the transmission signal, to guarantee the unipolar character of the excitation generated from the Verasonics. On the other hand, to compensate for the cable load effect and to minimize the losses of the transmission line, the receive paths are buffered with unity gain operational amplifiers on the motherboard to provide impedance matching between the output of the ASIC and the Verasonics. The power and control signals are transferred via a flat ribbon cable from the motherboard to the daughterboard. An external FPGA (DE2-115, Altera Corp., San Jose, CA, USA) generates the control data to program the ASIC, and an external power supply provides the power for the daughterboard and the motherboard. The Verasonics computer controls the overall operation of the whole system through MATLAB (2014b, The MathWorks, Inc., Natick, MA, USA). 

### 2.5. Measurement Setup

#### 2.5.1. Electrical Characterization

The electrical performance of the ASIC and the whole signal chain from the ASIC to the Verasonics, including the cables and the motherboard, was evaluated by a test sample ASIC without the acoustic stack. Randomly selected element bond pads on the ASIC were wire-bonded to an externally accessible test pad on the daughterboard. The transmit, receive, power, and control bond pads on the ASIC were wire bonded to the daughterboard in the usual way. After programming the ASIC, we used an arbitrary waveform generator (AWG; 33250A, Agilent Technologies, Santa Clara, CA, USA) to apply a 50-cycle sinusoidal signal of 7.5 MHz to the test bond pad as a test signal. The corresponding output signals were recorded at three different locations: at the input of the buffer on the motherboard; at the output of the buffer on the motherboard (i.e., at the input of the Verasonics); and at the output of the analog-to-digital converter of the Verasonics. We present the electrical performance of the signal chain in Appendix A.

#### 2.5.2. Acoustical Characterization

**Figure 6:**
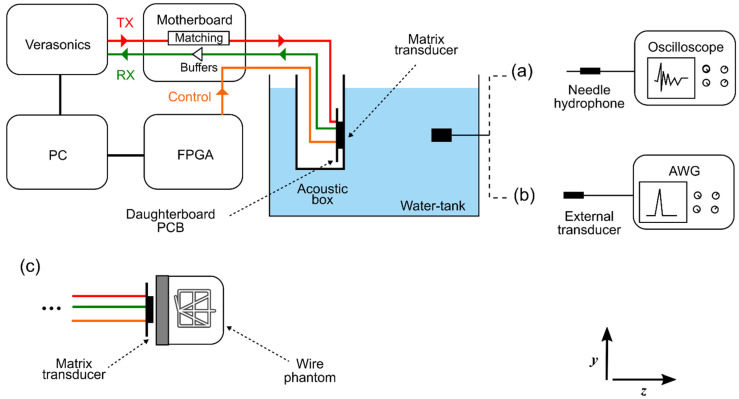
Acoustical measurement setup. (**a**) Transmit characterization. (**b**) Receive characterization. (**c**) Imaging using a CIRS phantom.

Figure 6 shows a schematic diagram of the setup for the acoustical evaluation of the prototype transducer. For this purpose, the transducer was mounted in a box with an acoustical window made of 25 μm thick polyimide. The whole setup was submerged in a tank filled with deionized water.

For the transmit characterization (see option (a) in Figure 6), each element was driven individually with a 20 V unipolar pulse provided by the Verasonics imaging system. The acoustic pressure generated by the elements was detected by a calibrated 1 mm needle hydrophone (SN2082, Precision Acoustics Ltd., Dorchester, UK) positioned at a distance of 200 mm away from the transducer in the z-direction. On the xy-plane, the hydrophone was placed in front of the active elements in order to reduce the influence of its directivity, as follows. In the y-direction, we aligned the hydrophone with a central row of the ASIC under testing, whereas in the x-direction, the hydrophone was aligned at two different positions: at column 20 for measuring elements located on the left-hand side (i.e., columns 1 to 40) of the matrix shown in Figure 5; and at column 60 for measuring elements at the right-hand side (columns 41 to 80). The hydrophone output was amplified by a 60 dB amplifier (AU-1519, Miteq, Inc., Hauppauge, NY, USA), digitized by an oscilloscope (DSO-X 4024A, Agilent Technologies, Santa Clara, CA, USA), and automatically transferred to the Verasonics computer. Lastly, the recorded signals were bandpass filtered with cutoff frequencies of 4 and 12 MHz to eliminate noise from lower and higher frequency sources.

The directivity pattern of nine arbitrarily selected elements was characterized in the x- and y-directions with hydrophone scans. For this purpose, we used a calibrated 0.2 mm needle hydrophone (SN2385, Precision Acoustics Ltd., Dorchester, UK) located 50 mm away from the transducer in the z-direction. The hydrophone output was amplified and recorded as detailed in the previous paragraph. For comparison, we have also simulated the directivity pattern of an equivalent rectangular piston using the ultrasound simulator FOCUS [50]. The relevant simulation parameters are given in Table 1.

To evaluate the receive performance (see option (b) in Figure 6), an external transducer was utilized as a transmitter and excited with a 3-cycle sinusoidal burst generated by an AWG. We used a pre-calibrated 1 mm circular single-element transducer (PA865, Precision Acoustics Ltd., Dorchester, UK), which was placed at the center of the matrix’s surface (i.e., at the origin of the xy-plane) at a distance z = 300 mm. The received signals of each individual element were acquired with the Verasonics.

Using the same setup described in the previous paragraph, we have also measured the overall dynamic range, which is defined as the difference between the highest and the lowest detectable pressures. With input pressures ranging from about 1 Pa to 50 kPa, we recorded the received signals for different gain settings of the Verasonics time-gain-compensation (TGC) and the ASIC gain (i.e., the combinations of LNA/PGA gain settings). For the ASIC gains, we used gain levels of 0, 3, 7, 11, and 15, which in decibels correspond to −8.6 dB, 0 dB, 12.1 dB, 23.5 dB, and 32.7 dB, respectively. 

#### 2.5.3. Imaging

To test the imaging capabilities of the transducer prototype (see option (**c**) in Figure 6), we imaged a commercial ultrasound phantom for 3D evaluation (model: 055, CIRS, Inc., Norfolk, VA, USA), which contains wires with a diameter of 100 µm. For this test, only the top ASICs (i.e., ASICs 1 to 4) of the prototype were active, which means about 3800 active elements. All the active elements were excited simultaneously to generate a plane wave. In reception, the echoed wavefronts were recorded column-by-column. This necessitates 80 transmit events for recording the data detected by all elements. The raw echo data was filtered using a 50th-order bandpass finite impulse response (FIR) filter with cut-off frequencies of 5 and 9 MHz. The sound speed value used for the reconstruction was 1540 m/s. 

A 3D volume with a lateral/elevation size of about 14 mm covering a depth ranging from 5 to 35 mm was discretized with a pixel size of 100 µm and reconstructed with a delay-and-sum beamforming technique. The reconstruction was conducted using a GPU code developed based on the direct sampling concept [51,52]. The beamformed echo data were first normalized, then log-compressed, and finally, shown with a dynamic range of 50 dB and 30 dB for 3D and 2D representation, respectively. The 3D volumetric image rendering was performed with MATLAB. For quantitative evaluation, the full width at half-maximum (FWHM) of the point spread function (PSF) from wire reflections in the lateral and axial directions was calculated in different elevation planes (from −3 mm to 3 mm, with a spacing of 1 mm) to evaluate the variability of resolution. 

## 3. Results

### 3.1. Sensitivity

**Figure 7:**
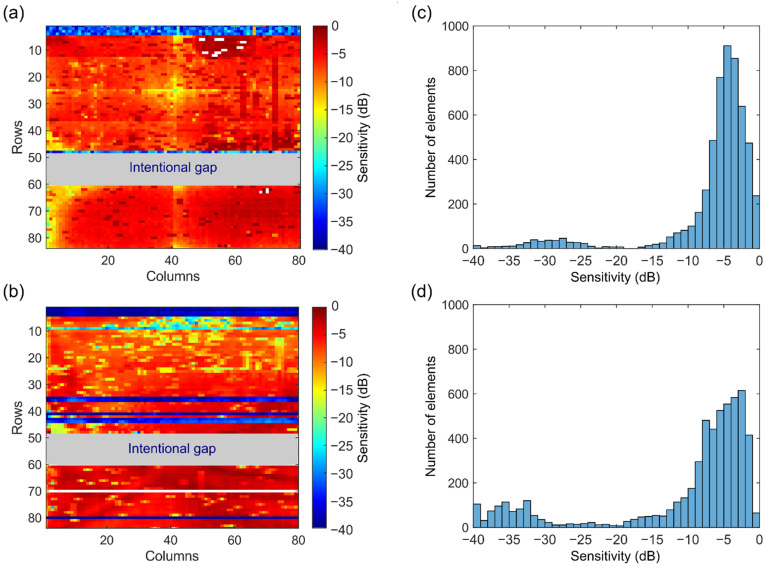
Sensitivity variation across the transducer elements. (**a**) Transmit sensitivity. (**b**) Receive sensitivity. (**c**) Transmit sensitivity histogram. (**d**) Receive sensitivity histogram.

The normalized sensitivity map across all elements of the matrix transducer in transmit and receive are presented in Figure 7a,b, respectively. The plotted values represent the envelope peak of the time domain signals expressed in decibels (relative to the maximum). Note that some elements (shown in white) have been omitted from the map because they exhibit a considerably higher amplitude (10 dB higher than the mean amplitude over all elements), which hinders the visualization of the variation across the remaining elements. Note also that ASICs 7 and 8 have suffered damages during the fabrication process and have been disconnected from the daughterboard. Due to this, we cannot include these ASICs in the overall evaluation of the prototype transducer, and we have omitted them in all figures presented in this section.

The sensitivity variation is somewhat similar in transmit and receive, but there are noticeable differences between them. In transmit, the elements located at the left bottom corner of the matrix (i.e., near columns 1 to 4 from ASICs 4 to 6) and also the ones located near the vertical centerline (i.e., near column 40 from ASICs 1 to 6) exhibit a lower efficiency, which is below −10 dB. The measurements for these elements were likely affected by the directivity of the hydrophone, as this effect is not seen in the receive map. In total, five rows are not functioning in transmit: rows 1 to 4, and row 48. This means that 400 elements are defective in transmit, which corresponds to 7% of the elements if we consider only elements from ASICs 1 to 6. In receive, however, there are significantly more defective rows: 12 in total. This represents 960 elements, i.e., 17% of the elements from ASICs 1 to 6. Besides the defective rows, many elements in ASICS 1 and 2 show a lower sensitivity (below −10 dB) in receive. Further observations regarding defective elements will be presented later in the Section 4.

Figure 7c shows the histogram of transmit sensitivity. As can be seen, about 3900 elements are within the 0 dB to −6 dB level. This represents 72% of the elements if we consider only functioning elements (i.e., elements located in defective/missing rows are not counted). In receive, as shown in the histogram in Figure 7d, about 2800 elements have a sensitivity above −6 dB. This represents 58% of the functioning elements.

### 3.2. Time and Frequency Response 

**Figure 8:**
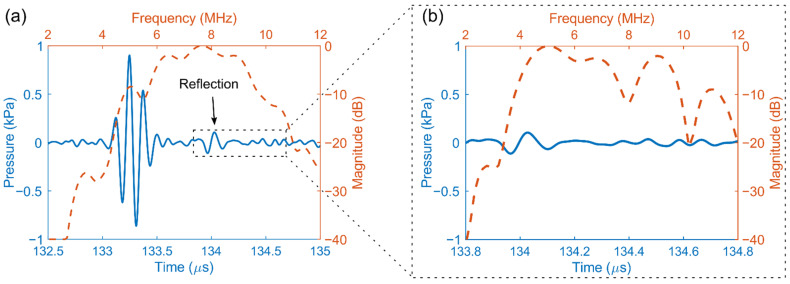
(**a**) Time and frequency domain response for a single element. (**b**) Close-up look at the second pulse.

Figure 8a shows the transmit pressure wave for a single transducer element (blue solid line), recorded with the hydrophone at z = 200 mm, together with its fast Fourier transform (red dashed line). Note that there is a second pulse present in the time domain response, with a delay of about 1.5 µs from the main pulse (see black arrow in the figure). This is likely due to reflections from the back side of the ASIC. The second pulse exhibits a peak frequency of about 5 MHz, as seen in the frequency spectrum in Figure 8b.

**Figure 9:**
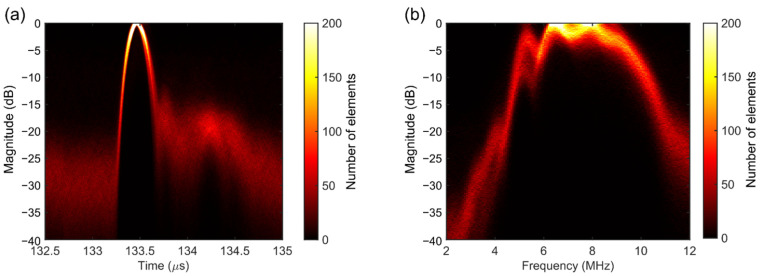
Time and frequency domain responses for all elements. (**a**) Envelope of the time signals. (**b**) Frequency spectrum.

In Figure 9, the time and frequency responses for all working elements of the transducer are presented. In the figure, the color of each pixel represents the count of the number of occurrences in that pixel. In the time domain response shown in Figure 9a, the time delays between the signals have been corrected using cross-correlation.

The transmit performance of the transducer is summarized in Table 2 in terms of peak pressure, center frequency, −6 dB bandwidth, and ringing time, which is defined as the time interval for the envelope amplitude to decrease below −20 dB of its corresponding peak. The listed values represent the mean and standard deviation over the working elements (i.e., rows 1–4 and 48 are neglected in the calculations).

### 3.3. Directivity Pattern

**Figure 10:**
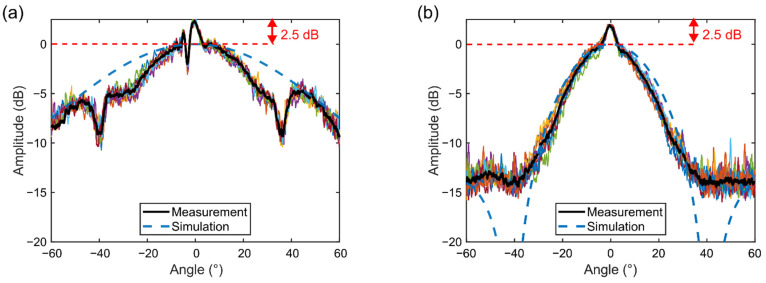
Measured and simulated directivity pattern in transmit. (**a**) Along the x-direction. (**b**) Along the y-direction.

Figure 10 shows the measured directivity pattern of nine arbitrarily selected elements in transmit together with the corresponding averaged directivity pattern (black solid line) and the simulated result (blue dashed line). The measured directivity pattern differs somewhat from the simulated one in both directions. As indicated in the figure, the measurements show a sharp peak with an amplitude of 2.5 dB higher than in simulation at zero degrees. Ignoring this sharp peak, the directivity pattern along the x-direction exhibits a −6 dB beam width of about 70 and 105 degrees in measurements and simulations, respectively. This difference is due to the presence of a dip at ±40 seen in the measurements. In addition, an extra dip at −4 degrees is also present in the measurements. Along the y-direction, the measured directivity pattern shows a −6 dB beam width of about 42 degrees, which agrees well with the simulated one. The dips observed in the simulation at ±40 degrees are not present in the measurements due to the noise floor, which is about −14 dB. 

### 3.4. Dynamic Range

**Figure 11:**
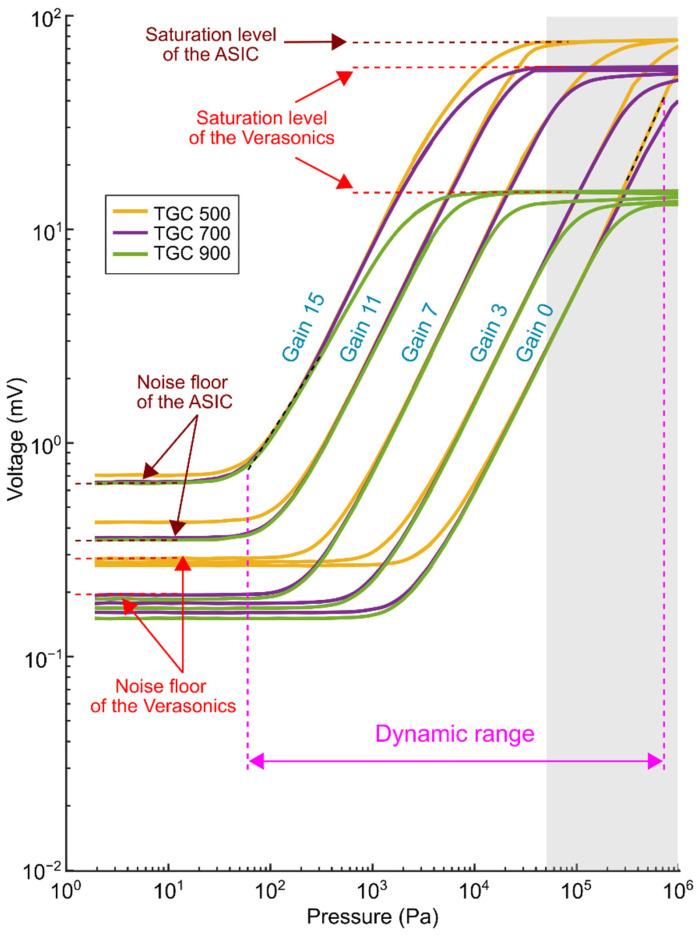
The relation between the received pressure and ASIC output voltage for different gain settings.

Figure 11 shows the relation between the received pressure at the transducer surface and the corresponding ASIC RX output voltage for different TGC and ASIC gain settings. The plotted values represent the average over all the functioning elements. The received signal amplitudes were converted from Verasonics output units back to millivolts at the Verasonics channel input using the results obtained from the electrical characterization (see Appendix A). Note that the values ranging from 50 kPa to 1 MPa (the gray region in the figure) were extrapolated based on the trend before and the observed saturation limits. This was done because we did not apply pressures above 50 kPa to avoid damage to the transmitting transducer. 

It can be seen that the relationship between received pressure and the output voltage is characterized by both linear and non-linear regimes. In the mid-range, where the curves are nearly linear, we observed an average difference of about 3 dB between most of the adjacent ASIC gain steps. For the lowest gain, we measured a receive sensitivity of approximately 55 nV/Pa, whereas for the highest gain the receive sensitivity is about 9 µV/Pa. This corresponds to a total gain range of 44 dB. 

In the low range, the curves are dominated by the noise floor of both the Verasonics and the ASIC, as indicated in the figure. It is noticeable that the noise floor varies for different TGC and ASIC gain settings. As can be seen, the noise floor for higher ASIC gain levels (gains 11 and 15) remains approximately the same regardless of the TGC changes. From this perspective, we can conclude that the noise floor for higher ASIC gain levels is determined exclusively by the ASIC gain. On the other hand, for lower ASIC gain levels (gains 0 to 7), the noise floor remains basically the same regardless of the ASIC gain changes. Therefore, we can say that the noise floor for this gain setting is determined by the Verasonics only. Note that TGC 500 exhibits a slightly higher noise floor than TGC 700 and 900, possibly because the noise floor is significantly determined by both the ASIC and the Verasonics.

**Figure A1:**
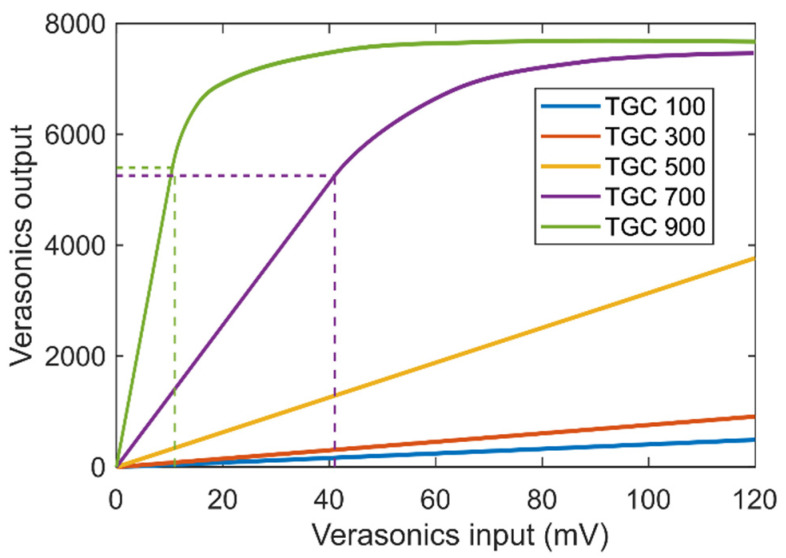
Relation between the input and output of the Verasonics V1 system for several TGC gains at 7.5 MHz.

In the high range, the curves are dominated by the saturation levels of the Verasonics and the ASIC. As noticed in the figure, the saturation level for TGC 900 is about 15 mV, whereas for TGC 700 it is 60 mV. This is in agreement with the corresponding saturation levels presented in Appendix A. However, for TGC 500 we did not observe a saturation at about 80 mV in the electrical characterization (see Figure A1 in Appendix A). Therefore, the saturation observed in Figure 11 for TGC 500 actually corresponds to the saturation level of the ASIC (note that the saturation level of the ASIC is irrespective of the ASIC gain). Because of the observed saturation values for both the ASIC and Verasonics, we were able to extrapolate the results above 50 kPa.

The receive performance of the prototype transducer for different gain settings is summarized in Table 3. Here, the minimum detectable pressure is defined as the pressure level at which the SNR becomes 0 dB, whereas the maximum detectable pressure is defined as the pressure level at which the 1 dB compression is reached.

### 3.5. Imaging

**Figure 12:**
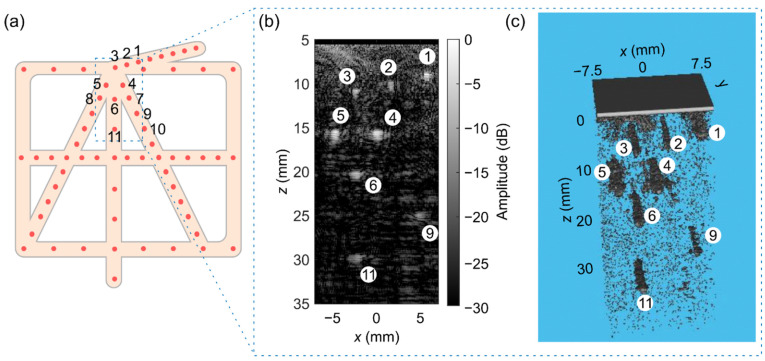
(**a**) Scheme of the wire phantom and numbered wires (the dashed rectangle depicts the field of view of the transducer). The reconstructed (**b**) 2D and (**c**) 3D images.

**Figure 13:**
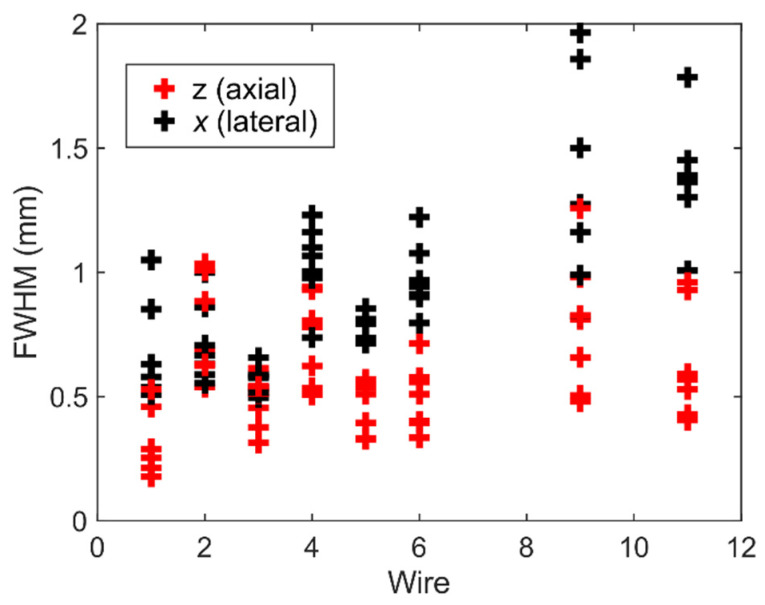
The axial and lateral FWHM for different wires in different elevation planes.

Figure 12 shows the schematic representation of the wire phantom together with the reconstructed 2D and 3D images (the 2D image is one slice of the 3D image). As can be seen, the wires numbered 1 to 6 and 11 are clearly detectable in both the 2D and 3D images. Wires 7, 8, and 10 were not detected though, and wire 9 was barely detectable. This could be due to the small effective aperture (contribution of a low number of elements) in reconstructing the pixels on the edge of the image. The trend of lateral FWHM in Figure 13 indicates that the lateral resolution degrades when the imaging depth increases. The range of lateral and axial FWHM is almost the same for all the wires except wires 9 and 11, which are positioned at larger z values. The wide range of FWHM values for each wire mainly comes from the different sensitivity of the elements of the prototype transducer.

## 4. Discussion

In this work, we have presented a 7.5 MHz prototype transducer for 3D imaging of the carotid artery. We have built an array of 8 × 1 tiled ASIC integrated with a PZT matrix consisting of 7680 elements. We have opted to leave a gap in the middle of the array to reduce the risk of mechanical damage to the ASICs during the manufacturing process due to misalignment. The current size of the gap is one full ASIC due to the current design of the daughterboard PCB layout, but if building a large aperture in parts remains necessary, a redesigned PCB can reduce the gap to a single row. Unfortunately, two ASICs still were malfunctioning due to electrical issues: in ASIC 8, we observed a short in one of the power supplies to the ASIC during manufacturing and decided not to use this ASIC further. In ASIC 7, we found a short during the final check before finishing the fabrication and decided to remove the bond wires for this ASIC. We are currently investigating ways to minimize damage to the ASICs (both mechanical and electrical) during the fabrication process and increase the element yield. One potential approach is to prefabricate the acoustic stack (i.e., the PZT matrix, matching layer, and interposer) separately and attach it to the ASIC pads (or gold balls) at a later time. The procedure of bonding the acoustic stack to the ASIC could be accomplished by using an anisotropic conductive film, as described in [39]. In addition, the risk of electrical damage, such as caused by electrostatic discharge (ESD) events, could be significantly reduced if we are able to effectively ground leakage paths during the assembly of the acoustic stack on the ASICs. We will explore these possibilities in our future work.

The maps presented in Figure 7 show that 72% and 58% of the working elements exhibit sensitivity variation within the −6 dB range in transmit and receive, respectively. On the one hand, the achieved element yield is sufficient to demonstrate the technology employed in the prototype transducer, allowing us to evaluate features and test the functionality of the current design. On the other hand, for imaging purposes or mass production, the element yield must be improved to avoid defective rows. This is a major problem that needs to be tackled in our manufacturing process. Regarding rows 1 to 4, which do not work in both transmit or receive, we found afterward that this was caused by a damaged cable in our measurement setup. Regarding the rows that do not work only in receive (mostly from ASIC 4), we believe this is due to faulty wire bonds or damage to the motherboard components. Besides defective rows, many elements in ASICs 1 and 2 show a lower sensitivity (below −10 dB) in receive. These elements probably suffered damage/degradation during or after the transmit experiments (the transmit and receive measurements were performed in an interval of one week). The degree of degradation might be verified by repeating the transmit characterization and comparing it with the previous measurements. Regarding the omitted elements in the map (shown in white), we think that they exhibit a considerably higher amplitude due to a short in the acoustic stack between multiple elements. 

Figure 8 and Figure 9 show that the time and frequency responses of different transducer elements are quite similar (based on the number of overlapped pixels in Figure 9) and behave as expected. Based on the measured peak pressure (0.6 kPa) and the transmit voltage (20 V peak amplitude), we estimate an average transmit efficiency of approximately 30 Pa/V at 200 mm. This value is comparable with our previous prototype with subdiced elements [44]. On average, the elements have a center frequency of 7.5 MHz and a −6 dB single-way bandwidth of about 45%. However, we have observed that many elements exhibit a sharp peak at 5 MHz and a dip at 6 MHz, which reduces their bandwidth significantly. This is likely caused by the effect of reflections and standing waves from the bottom side of the transducer, i.e., from the ASICs. 

The measured directivity pattern shown in Figure 10 follows the trend of the simulated one in both directions but deviates significantly at specific points. These deviations can be explained by a combination of both electrical and acoustical crosstalk (see Appendix B for details). The electrical crosstalk in our case means that all elements of a row are somewhat excited when an electrical pulse is sent to the transmit bus of that row. This is likely the cause of the sharp peak of 2.5 dB at 0 degrees. Since this type of crosstalk only happens in transmit, the sharp peak will be absent in the receive directivity pattern. Furthermore, because we intend to use at least half of the elements on a row in transmit, the peak will not affect the images generated by this probe. Along the x-direction, the dips observed at ±40 degrees are likely caused by acoustical crosstalk. Since the prototype transducer was designed to operate with low steering angles, these dips are not considered to be important. Previously, in the design without an interposer layer, we also observed peaks at ±20 degrees in the directivity pattern. With the interposer, there is now an attenuating medium in between the elements and the ASIC and due to that, these peaks do not show up anymore. This suggests that the employed interposer layer helps to reduce the crosstalk due to the propagation of Lamb waves in the ASIC. 

As seen in Figure 11, the minimum detectable pressure of 60 Pa is limited by the noise floor of the ASIC for gain 15. On the other hand, the maximum detectable pressure is about 700 kPa, which is limited by the saturation level of the ASIC for gain 0 and TGC 500. Therefore, the overall dynamic range of the prototype transducer is about 81 dB, which is sufficient for carotid imaging applications [53].

The performance of the prototype transducer was tested by imaging a commercial wire phantom, as shown in Figure 12 and Figure 13, which proves the applicability of the prototype for plane wave 3D imaging. Future work should include further evaluation of beamforming image quality and in vitro and in vivo experiments.

## 5. Conclusions

We have demonstrated the design, fabrication, and characterization of a PZT matrix transducer with integrated electronics. The ASIC architecture together with the subdicing of the piezo elements allowed us to effectively reduce the channel count to 120 transmit and 120 receive channels. The prototype transducer was targeted to have 7680 elements built on top of 8 × 1 tiled ASICs; however, two ASICs were damaged during the fabrication process. On average, the individual elements of the transducer exhibited a transmit efficiency of 30 Pa/V at 200 mm and a −6 dB bandwidth of 45%. The receive dynamic range is 81 dB with a minimum and maximum detectable pressure of 60 Pa and 700 kPa, respectively. Overall, the characterization results are promising and encourage us to pursue further up-scaling by fabricating a larger PZT matrix transducer on 10 × 1 tiled ASICs with an increased element yield. In this way, we expect to realize a fully populated matrix consisting of about 10,000 elements in the near future.

## Figures and Tables

**Table 1:** Parameters for the directivity pattern simulation.

Parameter	Value
Element center frequency	7.5 MHz
Element size	300 µm × 150 µm
Excitation type	Hanning weighted pulse
Number of cycles	1
Sound speed	1480 m/s

**Table 2:** Transmit performance of the prototype transducer.

Parameter	Value
Peak pressure (kPa)	0.6 ± 0.2
Center frequency (MHz)	7.5 ± 0.6
Bandwidth _−6 dB_ (%)	46 ± 14
Ringing time _−20 dB_ (µs)	0.3 ± 0.15

**Table 3:** Receive performance for different gain settings.

ASIC Gain	Minimum Pressure (kPa)	Maximum Pressure (kPa)	Receive Sensitivity (µV/Pa)
0	30	700 *	0.06
3	2	200 *	0.15
7	0.3	70 *	0.72
11	0.2	20	2.73
15	0.06	4	8.79

* Extrapolated values.

**Figure A2:**
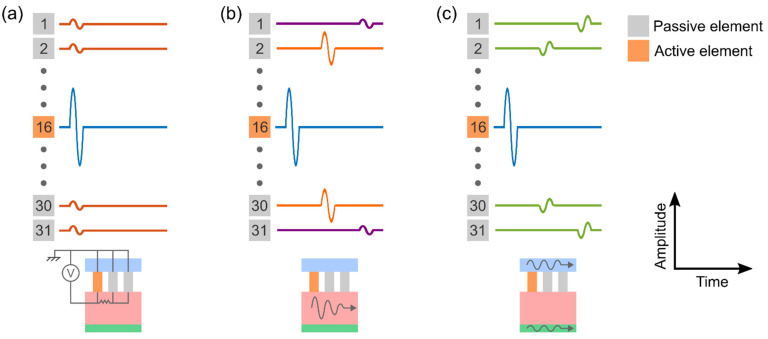
Assumptions for crosstalk simulations. (**a**) Electrical crosstalk. (**b**) Acoustical crosstalk via an attenuating medium. (**c**) Acoustical crosstalk via a non-attenuating medium.

**Figure A3:**
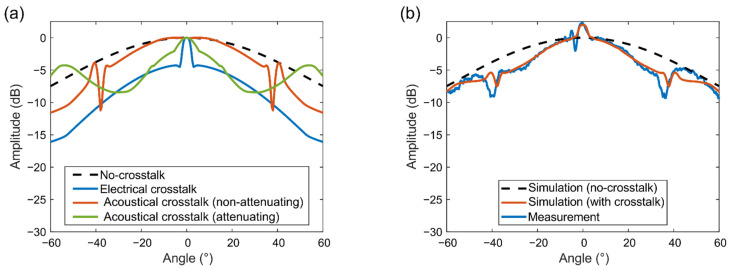
Directivity pattern along the x-direction. (**a**) Effect of individual crosstalks. (**b**) Effect of the combined crosstalk.

## Data Availability

The data presented in this study are available on request from the corresponding author.

## Appendix A. Electrical Characterization

Figure A1 shows the relationship between the output voltage of the ASIC (connected to the Verasonics input channel) and the output of the Verasonics for different TGC settings. The curves show that for TGC levels of 100, 300, and 500, the Verasonics signals are always linear in the range evaluated. However, with TGC levels of 700 and 900, nonlinear deformation of the signal is observed above 5500 units (shown by the dashed lines) due to the saturation of the Verasonics output. Table A1 presents the maximum acceptable input voltage (determined using the 1 dB compression point), the corresponding Verasonics output, and the gain in the linear range at each TGC setting. By increasing the TGC, the Verasonics gain is increased; however, the maximum acceptable input voltage in the linear regime is decreased.

**Table A1:** Characterization of the Verasonics V1 system for different TGC settings.

TGC	Maximum Input (mV)	Verasonics Output	Slope (1/mV)
100	310	1245	4.1
300	310	2315	7.6
500	185	5497	31.4
700	45	5514	129.3
900	11	5602	543.3

## Appendix B. Crosstalk Analysis

Herein, we investigate whether the differences observed between the simulated and experimental directivity patterns could be explained by interelement crosstalk present in the prototype transducer. Since our measurement setup and transducer configuration do not allow us to measure the crosstalk directly, we analyzed the possible effects of both electrical and acoustical crosstalk on the directivity pattern via simulations. For this, we used the ultrasound simulator FOCUS, as explained previously (see Table 1), to simulate the directivity pattern of the transducer elements excited with different amplitudes and time delays to mimic the crosstalk [54,55]. In all simulations, we used an array of 31 elements with the active element being the central one.

For the electrical crosstalk simulations, the neighboring passive array elements were excited simultaneously with an equal amplitude between them but a lower amplitude as compared to the active element, as shown in Figure A2a. With regard to the acoustical crosstalk, we hypothesized that there were two different kinds of acoustical crosstalk happening. First, we assumed that the vibration of the active element will generate wave propagation through the interposer layer that will induce a vibration in the neighboring elements. To simulate this, the passive elements were excited with amplitudes and delays based on the distance from the active element, as shown in Figure A2b. Second, we hypothesized that there will also be crosstalk via a non-attenuating medium such as the ASIC. Here the delays are again based on the distance from the active elements, but the amplitude of the passive elements is the same, as shown in Figure A2c. The crosstalk analysis was performed only along the x-direction.

Figure A3a shows the effect of each simulated crosstalk on the directivity pattern. As can be seen, the electrical crosstalk introduces a sharp peak at zero degrees. We observed that the magnitude of the sharp peak is determined by the amplitude of the passive elements. Regarding the acoustical crosstalk via the interposer, the beam is narrowed and two bumps appear, whose position is determined by the velocity of the medium. Finally, the acoustical crosstalk via the non-attenuating material generates two dips or peaks, depending on the phase.

To investigate the combination of electrical and acoustical crosstalk, we swept through different values of amplitudes and time delays in order to fit the simulated data to the experimental one. Figure A3b shows the result of the fitting procedure and Table A2 lists the values of amplitudes, time delays, and propagation speeds used to fit the curve. As seen, the trend of the simulated directivity pattern with crosstalk follows the experimental curve. This result suggests that the extra peak and dips observed in the measured directivity pattern could be caused by a combination of electrical and the two types of acoustical crosstalk. Note, however, that the value of propagation speed in the non-attenuating medium differs significantly from the propagation speed of Lamb waves in the ASIC [47], which suggests that this kind of crosstalk goes via another layer.

For our purposes, this brief analysis suffices to represent the contribution of different types of crosstalk in the directivity pattern. An in-depth simulation study of crosstalk is left for later work as this is beyond the scope of this paper.

**Table A2:** Parameters for crosstalk simulations.

	Electrical	Acoustical (Non-Attenuating)	Acoustical (Attenuating)
Amplitude	−30 dB	−20 dB	−3.5 dB *
Time delay *	-	0.0612 µs	0.0833 µs
Propagation speed	-	2450 m/s	1800 m/s

* Between adjacent elements.
